# EEG/MEG Source Imaging: Methods, Challenges, and Open Issues

**DOI:** 10.1155/2009/656092

**Published:** 2009-07-20

**Authors:** Katrina Wendel, Outi Väisänen, Jaakko Malmivuo, Nevzat G. Gencer, Bart Vanrumste, Piotr Durka, Ratko Magjarević, Selma Supek, Mihail Lucian Pascu, Hugues Fontenelle, Rolando Grave de Peralta Menendez

**Affiliations:** ^1^Department of Biomedical Engineering, Tampere University of Technology, 33101 Tampere, Finland; ^2^Department of Electrical and Electronics Engineering, Middle East Technical University, 06531 Ankara, Turkey; ^3^SCD, Department of Electrical Engineering (ESAT), Katholieke Universiteit Leuven, Kasteelpark Arenberg 10, B-3001 Leuven, Belgium; ^4^MOBILAB, Biosciences and Technology Department, Katholieke Hogeschool Kempen, Kleinhoefstraat 4, B-2440 Geel, Belgium; ^5^Department of Biomedical Physics, University of Warsaw ul. Hoża 69, 00-681 Warszawa, Poland; ^6^Faculty of Electrical Engineering and Computing, University of Zagreb, 10000 Zagreb, Croatia; ^7^Department of Physics, Faculty of Science, University of Zagreb, 10000 Zagreb, Croatia; ^8^National Institute for Laser, Plasma and Radiation Physics, Laser Department, 077125 Bucharest, Romania; ^9^Department of Medical Physics, School of Medicine, University of Patras, 26504 Patras, Greece; ^10^Electrical Neuroimaging Group, Department of Clinical Neurosciences, Geneva University Hospital, 1211 Geneva, Switzerland; ^11^Neurodynamics Laboratory, Department of Psychiatry and Clinical Psychobiology, University of Barcelona, 08035 Barcelona, Catalonia, Spain

## Abstract

We present the four key areas of research—preprocessing, the volume conductor, the forward problem, and the inverse problem—that affect the performance of EEG and MEG source imaging. In each key area we identify prominent approaches and methodologies that have open issues warranting further investigation within the community, challenges associated with certain techniques, and algorithms necessitating clarification of their implications. More than providing definitive answers we aim to identify important open issues in the quest of source localization.

## 1. Introduction

Electroencephalography (EEG) and magnetoencephalography (MEG) represent two noninvasive functional brain imaging methods, whose extracranial recordings measure electric potential differences and extremely weak magnetic fields generated by the electric activity of the neural cells, respectively. These recordings offer direct, real time, monitoring of spontaneous and evoked brain activity and allow for spatiotemporal localization of underlying neuronal generators. EEG and MEG share the following characteristics: (1) they have a millisecond temporal resolution; (2) potential differences and magnetic fields are linear functions of source strengths and nonlinear functions of the source support (e.g., dipole locations); (3) they are caused by the same neurophysiological events, that is, currents from synchronously activated neuronal tissue often referred to as the primary or impressed current source density, and thus both can be used equivalently for the localization of neuronal generators. 

The three-dimensional reconstruction of neural activity is commonly misconstrued as *tomography*, which is defined [1] as “*any technique that makes detailed predetermined plane sections of an object while blurring out the images of other planes*.” The physics governing the propagation of the electromagnetic fields depends on the composition of the volume conductor, which means that the source activity outside the predetermined plane also influences the readings of the sensors lying in the plane. So, actually the procedures applied in tomography and inverse solutions are somehow reversed: in tomography we reconstruct a 3D image by combining separately obtained slices, whereas, inverse solutions calculate the whole 3D distribution, which can be later presented as slices. While tomographic techniques (e.g., CT, PET, MRI, etc.) are associated with well-posed mathematical problems, the noninvasive estimation of the brain activity is essentially an ill-posed problem due to the infinite number of solutions. 

In the subsequent sections we remark about some important issues for the understanding, selection, and evaluation of source imaging methods; hence our emphasis is on general approaches rather than particular solutions. These sections reflect upon our group discussion held at a NeuroMath workshop. As a group we acknowledge that we have differences of opinion regarding the selection of methods, we face various challenges as separate research centers, and we differ on what are the key open issues due to our different interests. Therefore, we have attempted to write an article that benefits the novice, aligns disparate parts of the source imaging community, and focuses much needed attention to several open issues. 

### 1.1. Theory

The relationship between the sources inside the head and the external measurements *d* is described as

(1) d=Lj,

where *L* is the linear operator representing the lead field (also known as the gain model or the direct model), and *j* represents the sources. The two mathematical properties of (1) reflect the attributes of the physical magnitudes involved. Firstly, the homogeneity property states that the image of an amplified source *k* ∗ *j* is an amplified measurement *k* ∗ *d*, and secondly, the additive property states that the sum of the two sources *j* = *j*_1_ + *j*_2_ produces a measurement equal to the sum of each measurement alone *d* = *d*_1_ + *d*_2_. Together these two properties follow the superposition principle, that is, *L*(*k*_1_ ∗ *j*_1_ + *k*_2_ ∗ *j*_2_) = *k*_1_ ∗ *d*_1_ + *k*_2_ ∗ *d*_2_, where *d*_1_ = *Lj*_1_ and *d*_2_ = *Lj*_2_. 

The ill-posed nature of this problem arises from the fact that two different sources *j*_1_ and *j*_2_ might produce the same measurement *d*, that is, *d* = *Lj*_1_ = *Lj*_2_, which is trivially equivalent to say that there exists a “silent” source *h* such that *Lh* = 0. In order to see the equivalence, note that if *d* = *Lj*_1_ = *Lj*_2_, therefore, the silent source *h* = *j*_1_ − *j*_2_ fulfils *Lh* = *Lj*_1_ − *Lj*_2_ = *d* − *d* = 0. In the other direction, if we assume that *Lh* = 0 and the existence of source *j*_1_ such that *Lj*_1_ = *d*, we can always build a new source *j*_2_ = *j*_1_ + *h* that yields the same data, that is, *Lj*_2_ = *L*(*j*_1_ + *h*) = *Lj*_1_ + *Lh* = *Lj*_1_ = *d*. 

That being said, we can establish one of the main properties of EEG/MEG scalp distributions (maps). While similar scalp maps cannot rule out the possibility of different subjacent source distributions, different maps are necessarily due to different source distributions. Importantly, we do not need to resort to any inverse method to conclude that. 

Building on linearity and in the absence of a priori information to justify otherwise, we can represent the solution of (1) with a linear operator *G* that “estimates” *j* as follows:

(2) $$j_{\text{est}}=G*d.$$

Substituting *d* by its value defined in (1) yields a fundamental equation of linear operators relating to the estimated and the original source distribution

(3) $$j_{\text{est}}=G*L*j\;=R*j,$$

where *R* = *G* ∗ *L* is the resolution operator [2, 3]. In practice both the sensors and the geometry are made of discrete measurements, and thus it can be assumed that *L*, *G*, and *R* are finite dimensional matrices approximating the continuous (integral) operators.

## 2. Preprocessing

In this section, we discuss some relevant issues related to the preparation of the data identifying some useful preprocessing and things to avoid. In general, the philosophy of preprocessing is to prepare the signal for solving. Typically, these steps decompose complex signals and reduce the noise from the sensors as well as other undesirable sources.

**Figure 1:**
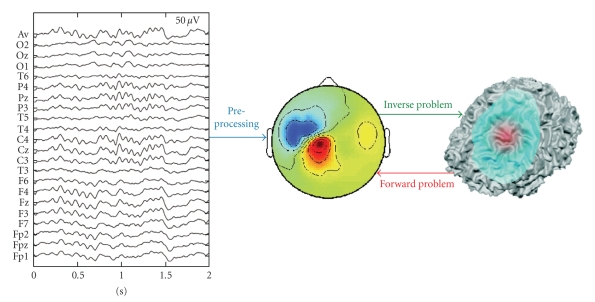
Key parts of source imaging. Preprocessing prepares the recorded signals for solving the inverse problem. The inverse problem attempts to locate the sources from recorded measurements, whereas the forward problem assumes a source definition in order to calculate a potential distribution map.

The EEG and MEG inverse problems (Figure 1, green arrow) start with the time series (Figure 1) recorded at the scalp sensors. Therefore, the localizations based on the distribution of scalp amplitudes in single time instants might be improved by the application of signal processing techniques to the measured time series (Figure 1, blue arrow). In particular the input noise can be reduced by selective and sensitive extraction of relevant activities from the EEG/MEG data. This can be achieved by localizing signal components extracted by a blind source separation (e.g., ICA [4]). Other approaches rely on the information derived from the time-frequency representations, corresponding to the relevant phenomena we want to localize (e.g., sleep spindles [5]). A similar but more sensitive and selective preprocessing was proposed in [6] using the multichannel matching pursuit algorithm. Overall, most preprocessing algorithms are expected to benefit the quality and accuracy of the inverse solution.

### 2.1. Epochs

We should weigh the advantages and disadvantages of the role epochs play in the recordings of event-related potentials. So far there has been no standard on the number of trials, jitter, averaging amplitudes, or the appropriateness of single trial analysis. For instance, the signal-to-noise ratio (SNR) increases with the number of trials, that is, the number of epochs; however, habituation can affect the results of some studies. We propose that a document outlining these categories would benefit future studies in terms of comparison and regularization.

The neuroelectric signals are buried in spontaneous EEGs with signal-to-noise ratios as low as 5 dB. In order to decrease the noise level and find a *template * Evoked Potential (EP) signal, an ensemble-average (EA) is obtained using a large number of repetitive measurements [7]. This approach [8] treats the background EEG as additive noise and the EP as an uncorrelated signal. The magnitudes and latencies of EP waveforms display large interindividual differences and changes depending on the psychophysiological factors for a given individual [9]. Consequently, one goal in the methodological EP research is to develop techniques to extract the true EP waveform from a *single sweep*. 

For clinical evaluations, either the template EP signal or possible amplitude and/or latency variations on single sweeps are used [9]. To observe such variations, the specific features are identified from a reference/template EP based on various estimation approaches. Since there are relatively tight constraints related to the available recording time or cooperativeness of the subject, the use of EA (as a reference EP signal) is usually impractical. This has led to the development of the alternative SNR improvement methods based on the additive model. Some of these algorithms are the weighted averaging approach, the subspace averaging method, the parametric filtering, the adaptive filtering, and Wiener filtering. In all these methods, it is assumed that the EP (i.e., signal) is stationary throughout the experiment. However, such assumptions are also questioned in some reports describing the event related potentials as superposition of some phase modulated rhythmic activities which may be related to different cognitive processes of the brain [10].

### 2.2. Things to Avoid

Contrary to the benefits of most preprocessing algorithms, there are certain algorithms that we should avoid before the application of source localization algorithms. In particular, the following choices threaten the integrity of the inverse solution.

(1) Baseline correction. Varying the values of individual electrodes either by “arbitrary” baseline shifting or by scaling factors changes the surface maps and thus the estimated sources. Although linear inverse solutions are rather stable (continuity with respect to the data), the application of base line correction to two conditions (that will be compared on the basis of their sources) can produce artificial differences induced by the correction and not by the real sources. 

(2) Artificial maps produced by grand mean data or segmentation algorithms. Statistical averages (e.g., mean) yield values that are usually not present in the original data. It would not be surprising if the average maps are not present in any of the subject averages. Furthermore, this effect can be amplified by the differences in latencies of the subjects. 

(3) The use of very high density of sensors might also jeopardize the source analysis due to different kinds of noise at different sensors. Moreover, no significant information is added after approximately 128 electrodes due to the noise levels. Lastly, some sensors might measure more artifacts than others due to their location near active muscles.

## 3. Volume Conductor

The head model as a volume conductor is a key element in source localization. The configuration of the volume conductor directly affects the solutions to the forward and inverse solutions. The three nearly equally important areas are head geometry, tissue conductivities, and electrode placement.

### 3.1. Geometry and Segmentation

The seminal study by Rush and Driscoll [11] used three concentric spheres, whereas, contemporary studies implement realistic models. We find that the differing models within the community are necessary, but how does each type of geometrical model contribute to the goal of source localization? The spherical models answer general questions of theory providing EEG localization accuracy of a few centimeters, while the realistic models attempt to pinpoint exact locations but actually improve dipole localization by a few centimeters [12–14]. On the other hand, the spherical geometry is sufficient for most MEG-based numerical simulations. Only the localization of the deep sources near the bottom of the skull in the frontotemporal and the frontal areas requires a realistically-shaped-head-volume conductor model for MEG-based simulations [15].

The geometry is directly related to the imaging modality, computed tomography (CT) or magnetic resonance imaging (MRI), and the quality of the segmentation. Naturally, we will question which modality to segment—CT, MRI, or fused CT-MRI images [16]. We are encouraging the modelers to understand the significance of the boundaries defined by a particular modality and are not in any way suggesting the medical community to provide any unsafe and unnecessary radiation to any patient. We must remember that the modality we select influences the segmentation due to its sensitivity to hard or soft tissues accordingly. Furthermore, how many tissues, which tissues, and which cavities should the models include? We foresee that we are nearing a plateau to the improvement in localization accuracy as our segmentation resolution increases along with the inclusion of too many small tissue regions. One avenue of research that could plausibly benefit from the development of the head-model geometry is the integration of anthropometric and craniometric data [17]. This path could justify individual models that claim to represent a subpopulation and repudiate studies that misrepresent an identified subpopulation beyond statistical significance. Moreover, it could lead us to establishing the statistical significance of the shape and size of the geometrical features within individual and groups of models.

### 3.2. Conductivity Values

The electrical characteristics of many biological tissues are inhomogeneous, anisotropic, dispersive, and nonlinear. Head tissues such as the skull, scalp, muscles, cerebrospinal fluid, and gray and white matter have different conductivities *σ*, permittivities *ε*, and magnetic permeabilities *μ* (in most cases it is considered equal to the permeability of water, which is in turn close to the permeability of free space *μ*_*o*_). The skull as well as the scalp has a multilayer structure, with each layer possessing different electrical properties. This fact leads either to multilayer modeling of the geometry of the tissue [18] or to attributing inhomogeneous properties to the tissue, that is, assigning tensors for *σ* = *σ*(*x*, *y*, *z*) and *ε* = *ε*(*x*, *y*, *z*). The values and distributions of inhomogeneities are an even more acute problem in patient populations where pathological processes are likely to significantly influence conductivities in affected brain regions. Could there exist an equivalent hybrid isotropic model that represents multiple anisotropy layers? How significantly would such approximations affect source localization within healthy individuals compared with patients with head pathologies?

The conductivity values of any model influence the lead fields of forward problems and the solutions of the inverse problems. Consequently, it is critical that we must assign as accurate conductivity values as reported from previous literature studies and extrapolate and interpolate the rest. As a community we have established electrical-property ranges for most head tissues in terms of conductivity *σ* and permittivity *ε*; however, we have to determine the actual electrical-conductivity distribution of an individual's head. As a result of these ranges, many historical studies assign average values to their tissues [15, 18–26]. Using an average value may not be appropriate for individualized models since those models may result in inaccurate solutions due to a function of position [27] or of age [28, 29]. Nevertheless, studies with patients [30, 31] have shown that using approximate conductivities ratios with an accurate geometrical description of the head (i.e., based on a subject's MRI) might yield reasonable, verifiable results for both cortical and deep EEG sources. Still, future models could benefit from using age-specific conductivities. We speculate that the application of age-based conductivity values applied to the skull tissues—most especially the trilayer skull tissue—would mostly benefit the models of youth, whose ossification centers change rapidly in the first two years and plateau in conductivity value around 18 to 20 years of age when the calvarial ossification process is completed [32].

In order to solidify our motivation for highlighting the significance of the skull conductivity, we must briefly delve into its history. The pioneering work [11] introduced a standard conductivity ratio for the brain-to-skull-to-scalp of 1:80:1, which is a historical value still used by some researchers over four decades later. In [33] are reported measurements on postmortem cadavers yielding a ratio of brain-to-skull conductivity values of 15:1. Three years later [28] presented conductivity values on live tissue as low as a ratio of 4:1. Subsequently, Wendel and Malmivuo [29] correlated *postmortem * to live tissue measurements as a way to incorporate and evaluate past data due to the lack in live tissue measurements. Their previous work used a scaling ratio of 0.33 to 0.4 to accommodate the change in conductivity from living to *postmortem * tissue based upon the conductivity recordings of dying tissue samples [34, 35]. In that previous paper they presented an open issue to the community to make more measurements on live tissue samples—most especially live skull samples—at normal body temperature and moisture, which still remains as an open issue today. Therefore, it is pertinent that we discriminate the conditions under which tissue conductivity and permittivity values were and will be acquired. Values obtained by in vivo or living in vitro measurements should be preferred over *postmortem * measurements. In the case of *postmortem* measurements, the time and temperature of acquisition should be specified since tissue properties change rapidly after cellular death.

### 3.3. Acquisition of Conductivity Values

In the last two decades, a number of approaches have been proposed to image the electrical conductivity of the human body. In conventional Applied Current Electrical Impedance Tomography (ACEIT) low-frequency-sinusoidal currents are applied via electrodes attached to the body surface [36]. In Induced Current Electrical Impedance Tomography (ICEIT), time-varying magnetic fields with different spatial-field patterns are applied to induce current in the body. In both cases, surface electrodes are used to make voltage measurements. 

Recently, two new approaches were proposed that utilize magnetic measurements in determining the conductivity distribution. In Magnetic Induction Imaging (MII), a transmitter coil is driven by a sinusoidal current to provide time varying magnetic fields [37, 38]. When a body is brought nearby these coils, eddy currents are induced in the body. The distribution of these currents is a function of the body's conductivity distribution. These currents create secondary magnetic fields, and the electromotive force induced in a receiver coil is measured. In Magnetic Resonance Electrical Impedance Tomography (MR EIT), low-frequency currents are applied from the body surface, and the resulting magnetic fields are measured using an MR system [39, 40]. Since magnetic fields are measured inside the body, high-resolution images can be measured. Note that all methods are still in the investigation phase, and none of them can provide the requirements of high-resolution conductivity information required for source localization.

### 3.4. Electrode Montages

EEG has been traditionally measured using the standard 10–20 electrode system including only 21 measurement electrodes. It has been widely acknowledged that the spatial resolution of the 10–20 system is not sufficient for modern brain research [41–44]. The first step in improving the spatial resolution of EEG is to increase the number of EEG electrodes, which the market has responded to with commercially available systems including up to 256 electrodes.

During the last two decades several studies have investigated the benefits of increasing the number of EEG electrodes. The effect on the accuracy of both the forward solutions and inverse solutions has been evaluated. In several articles, an increase in the number of electrodes to at least 128 has been shown to improve the accuracy of the results [45–50].

Different factors affect the appropriate number of electrodes. These include, for example, the widely debated value of the skull's relative conductivity, which has a great impact on the accuracy of inverse solutions. Additionally, the spatial resolution of especially the dense EEG systems (128–512 electrodes) is extremely sensitive to measurement noise. Thus, for different EEG measurements conducted in different environments, the appropriate number of electrodes may vary considerably [48]. Using active electrodes will reduce the noise.

## 4. Forward Problem

The 1969 study by Rush and Driscoll [11] on EEG electrode sensitivity ushered in the new era of source localization. Their work analytically solved Maxwell's equations to map the lead field, which is only possible with at least elliptical symmetry. Contemporary models consist of a combination of complex geometry and/or electrical parameters, thus necessitating numerical solutions such as the boundary element method (BEM), finite element method (FEM), and the finite difference method (FDM) (Table 1). In this section we aim to identify some of the complications, advantages, and disadvantages of these numerical methods. Through the following explanations we hope the reader gains an understanding of the differences presented, adopts one or more appropriate methods specific to his/her requirements, and refers to the references for specific information.

Most models are unable to obtain the direct solution so they rely upon iterative solvers such as the successive over-relaxation (SOR), conjugate gradients (CG), preconditioned conjugate gradient method (PCG), and algebraic multigrid (AMG) solvers. While these methods have been developed for regular linear systems, they can also be applied in our semidefinite case. In the case of a consistent right-hand side, semiconvergence can be guaranteed for SOR and (P)CG, while the AMG theoretical results are more complicated [51]. A summary of each method is given based on [52] for the first three methods and [53, 54] for the last method.

A first difference between BEM and FEM or FDM is the domain in which the solutions are calculated. In the BEM the solutions are calculated on the boundaries between the homogeneous isotropic compartments while in the FEM and FDM the solution of the forward problem is calculated in the entire volume. Subsequently, the FEM and FDM lead to a larger number of computational points than the BEM. On the other hand, the potential at an arbitrary point can be determined with FEM and FDM by interpolation of computational points in its vicinity, while for the BEM it is necessary to reapply the Barnard formula [55] and numerical integration.

Another important aspect is the computational efficiency. In the BEM, a full matrix (**I** − **C**), represented in

(4) $$V=CV+\;V_{0},$$

needs to be inverted. When the scalp potentials need to be known for another dipole, **V**_0_ in (4) needs to be recalculated and multiplied with the already available (**I** − **C**)^−1^. Hence once the matrix is inverted, only a matrix multiplication is needed to obtain the scalp potentials. This limited computational load is an attractive feature when solving the inverse problem, where a large number of forward evaluations need to be performed. Alternatively, an accelerated BEM approach increases the speed considerably by calculating only *m* (i.e., the number of electrodes) rows of the corresponding inverse, whereas, the normal inversion process requires a lot more time due to the dimensionality of the matrix as *n* × *n* (i.e., *n* equals the number of nodes) [56, 57]. Projective methods [58] based on the parametric representation of the surfaces also allow for a drastic reduction of the computational load. 

For the FEM and the FDM, a direct inversion of the large sparse matrices is not possible due to the dimension of the matrices. Typically at least 500 000 computational points are considered thus leading to system matrices of 500 000 equations with 500 000 unknowns, which cannot be solved in a direct manner with the computers currently available. However, matrices found in FEM and FDM can be inverted for a given source configuration or right-hand side term, utilizing iterative solvers such as the successive over-relaxation method (SSOR), the conjugate gradient (CG) method [59], or algebraic multigrid (AMG) methods [60, 61]. A disadvantage of the iterative solvers is that for each source configuration the solver has to be reapplied. The FEM and FDM would be computationally inefficient when an iterative solver would need to be used for each dipole. To overcome this inefficiency the reciprocity theorem is used [62].

When a large number of conducting compartments are introduced, a large number of boundaries need to be sampled for the BEM. This leads to a large full system matrix, thus lower numerical efficiency. In FEM and FDM modeling, the heterogeneous nature of realistic head models will make the stiffness matrix less sparse and badly conditioned. Moreover, the incorporation of anisotropic conductivities will decrease the sparseness of the stiffness matrix. This can lead to an unstable system or very slow convergence if iterative methods are used. To obtain a fast convergence or a stable system, preconditioning should be used. Preconditioning transforms the system of equations *Ax* = *b* into a preconditioned system *M* ^−1^ *Ax* = *M* ^−1^ *b*, which has the same solution as the orignal system. *M* is a preconditioning matrix or a preconditioner, and its goal is to reduce the condition number (ratio of the largest eigenvalue to the smallest eigenvalue) of the stiffness matrix toward the optimal value of 1. Basic preconditioning can be used in the form of Jacobi, Gauss-Seidel, Successive Over-Relaxation (SOR), and Symmetric Successive Over-Relaxation (SSOR). These are easily implemented [63]. More advanced methods use incomplete LU factorization and polynomial preconditioning [63, 64].

For the FDM in contrast with the BEM and FEM, the computational points lie fixed in the cube centers for the isotropic approach and at the cube corners for the anisotropic approach. In the FEM and BEM, the computational points, the vertices of the tetrahedrons and triangles, respectively, can be chosen more freely. Therefore, the FEM can better represent the irregular interfaces between the different compartments than the FDM, for the same amount of nodes. However, the segmented medical images used to obtain the realistic volume conductor model are constructed out of cubic voxels. It is straightforward to generate a structured grid used in FDM from these segmented images. In the FEM and the BEM, additional tessellation algorithms [65] need to be used to obtain the tetrahedron elements and the surface triangles, respectively, although cubic and rectangular prism elements are possible in FEM like FDM.

Finally, it is known that the conductivities of some tissues in the human head are anisotropic such as the skull and the white matter tissue. Anisotropy can be introduced in the FEM [66] and in the FDM [67], but not in the BEM.

## 5. Inverse Problem

While previous sections focused on the different steps preceding the application of inverse procedures, that is, head geometry approximations, conductivity, geometry profile, accuracy of conductivity values, and so forth, this section discusses some open issues including the selection of the recording modality, the source model, and possible post processing to improve the robustness of the inverse solution estimates. 

### 5.1. Recording Modality: MEG versus EEG

The introduction of the Superconducting Quantum Interference Device (SQUID) made it possible to measure the very low magnetic fields induced by the electric activity of the brain, called magnetoencephalography, MEG. 

In the beginning of biomagnetic research, there was a lot of hope that biomagnetic signals would include information independent on the bioelectric signals. As described by Plonsey [68], the fact that according to the Helmholtz theorem the scalar and the vector potential fields could be selected independently was considered as evidence for the independence of the electric and magnetic measurements. On the other side, considering the origin of the bioelectric currents it is concluded that the divergence and the curl of the primary current could not be really arbitrarily assigned. Further experiments described in [68] pointed to the relevant contribution of the secondary sources to both electric and magnetic fields. Thus, while we cannot claim that measures of bioelectric or biomagnetic fields alone are enough to define the other [69], we should not expect important differences on the information recorded by them. 

The conclusion that electric and magnetic measurements provide comparable information has been confirmed on theoretical and simulation grounds. Using the novel concept of the half sensitivity volume, Malmivuo et al. [70] demonstrated that EEG and MEG record the electric activity in a very similar way, that is, the differences between the EEG and the MEG in the size of the half sensitivity volumes and the form of the sensitivity distributions are very small. Further evaluations of the spatial resolution for cortical sources in the spherical model led to better results for the EEG [71]. Using simulations [72] confirmed also a slight advantage of the EEG over many source locations and orientations with best results for the combined EEG/MEG measurements. More recently [73] applied pattern recognition techniques to decode hand movement directions from simultaneous EEG/MEG measurements, concluding that the inference of movement direction works equally well for both techniques. 

Therefore, it may be beneficial to consider also the cost effect of the recording modality. The MEG instrumentation costs about 20 times more than the EEG instrumentation with the same number of channels. Thus, for improving the accuracy of the inverse solution it might be beneficial to first improve all aspects of the EEG technology, that is, number of channels, electrode location accuracy, head model geometry, and tissue resistivity accuracy, and so forth, because improving all these cost much less than the MEG instrumentation.

**Figure 2:**
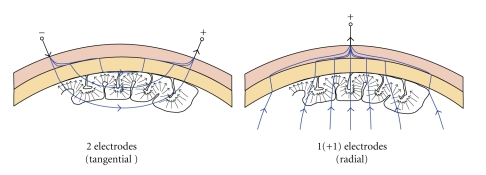
The Sensitivity Distributions of EEG. (Left) An EEG setup measuring the tangential components of neuroelectrical activity, where each bipolar lead is located relatively close to each other. (Right) An EEG setup measuring the radial components of neuroelectric activity, where the measuring electrode is located far from the reference electrode. The arrows in both figures represent macrocolumns of cellular architecture not dipolar sources.

**Figure 3:**
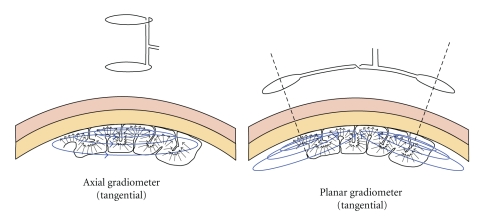
The Sensitivity Distributions of MEG. (Left) An MEG setup measuring the tangential components of neuroelectrical activity, using an axial gradiometer. (Right) An MEG setup measuring the tangential components of neuroelectric activity, using a planar gradiometer. The arrows in both figures represent macrocolumns of cellular architecture not dipolar sources.

In summary we can confirm to the reader that besides the cost differences, these two techniques offer similar information about brain sources in what concerns accuracy of source localization, spatiotemporal resolution, and decoding or predictive power. We would like to highlight that although similar information is detected, the EEG and MEG measurement sensitivities are orthogonal. The EEG primarily detects electric sources that are radial to the scalp surface with sufficiently distant electrodes and tangential components when the leads are located near to each other (Figure 2); however, the MEG primarily senses magnetic currents generated by electric sources in the radial direction (Figure 3). 

### 5.2. Source Models

There is vast literature reviewing the arsenal of methods available for the solution of the so-called bioelectromagnetic-inverse problem dealing with the estimation of the electrical activity (i.e., the distribution of sources) inside the head given external measurements of the electric and magnetic fields, for example, [44, 74, 75]. Nevertheless, before applying an inverse solution we must decide about the type of sources and their possible distribution (i.e., locations) inside the head. 

The inverse solution estimators differ in source modeling assumptions. By comparing the number of unknowns of the source model with the amount of data, we can differentiate two main type of problems (i.e., of solutions). Firstly, over-determined problems (e.g., dipolar solutions) with more data than unknowns can differ in minimization algorithms and their efficiency to escape local minima, measures of goodness of fit as well as the use of physiological and/or mathematical constraints often required in the solution estimation/selection process. These models require assumptions regarding the number and location of the brain sources modeled as point-current dipoles giving a unique solution provided that the global minimum is identified. Such approaches require a model order search in addition to a source parameter optimization [76]. Numerical simulation studies have demonstrated that an accurate estimation of the temporal dynamics of dipolar models is critically dependent on the ability to resolve and accurately localize all active brain regions [77]. While there is a range of physiological and anatomical reasons, animal studies as well as already converging evidence from the human hemodynamic and/or metabolic fMRI and PET studies suggest that the sensory and cognitive process can be considered as a network of distributed focal activity; possibility of extended activations of neuronal tissue in some conditions cannot be disregarded. In-depth electrode recordings used to be the primary evidence for the latter assumption, demonstrating activity over wide brain regions. However, even with such recordings, the summed contributions of the primary source contributions and volume currents are to be expected, and inverse models should be considered instead of taking such measures as strong evidence for extended brain activations.

On the other hand, we have the underdetermined problems (e.g., distributed inverse solutions) with more unknowns than data associated with the linear-minimum-norm approaches that is suggested [75, 78, 79] for cases when focal source assumptions are not justified. Such an approach is challenging as it might require further weighting and regularization to compensate for depth bias, selected by imposing mathematical criteria or physiological ones.

In order to help the reader make the correct choice, we describe four primary source models obtained by restriction on the source type and/or their location together with their main assumptions.

**(1) The equivalent-current dipole model**

It assumes that measurements are due to a single concentrated source. It is primarily valid for strong and spatially limited sources (e.g., some focal epilepsy) or sources observed from a far away measurement surface. It is probably more useful to summarize the measured field than the source itself, which is a particular case of the following source model.

**(2) Dipolar models as used in overdetermined problems**

These models consider that the measured fields are due to a small number of sources with unknown locations and orientations. They are very well suited for low-rank data as produced by filtered and averaged-evoked responses [80, 81].

**(3) Cortical model**

Under the extreme assumption that deep sources do not contribute to the external fields of the head, it assumes that the primary sources are located only in the cortical mantel with a direction constraint. It is probably very well suited for the analysis of measurements associated with the activation of some primary cortical areas [82].

Previous models can be considered as data driven in the sense that they can be only used under very specific and restrictive experimental conditions that will not be acceptable as a general model for the EEG and MEG sources. Furthermore, there is scarce experimental evidence in favor of the dipole. In fact a dipole would imply an indefinitely increasing potential when we approach its location. Hopefully, this has never been reported because that would correspond to an undefined potential at that location. Nevertheless, a more complete source model must contain, as a particular case, previous source models while incorporating those elements that are out of discussion so far, that is as follows.

**(4) Potential distribution inside the head**

The electromagnetic measurements at/near the scalp are due to the potential distribution inside the brain. These (intracranial) potentials that represent the primary source are generated in at, at least, the entire gray matter and not only at the cortex. This source model is compatible with all previous geometrical constraints while including the dipoles as a particular case. Importantly, this source model implies significant theoretical and numerical simplifications, solving also the issue of focal versus extended sources, since the potential is always a continuous function defined at all points of the head.

After defining the adequate preprocessing and source model for our data, we face the problem of the inverse procedure selection. The following issues might be relevant at this stage.

### 5.3. The Dipole Localization Error

The evaluation of the overdetermined-dipolar models seems to have a straightforward solution by comparing target and estimated sources with measures as the dipole localization error. Unfortunately, these measures cannot be directly extrapolated to under-determined distributed solutions. This is probably why the evaluation of the distributed solutions remains as an open issue in this field. Obviously, this might influence the selection of the inverse solution. While we do not want to tell the reader what he/she should do/use, we would like to discuss some things to avoid.

It has been suggested that the zero dipole localization might be the way to select the inverse solution. This is likely motivated by genuine applications where the data is dominated by single focal sources (e.g., epilepsy focus localization) as well as by the long experience accumulated from overdetermined (dipolar) models. It is probably an abuse of language, which brings people to believe that “if we correctly localize each single source alone, then by the principle of superposition we should correctly localize any combination of sources”. There are two clear inaccuracies with this statement.

1. In this case, correct localization only means that the maximum of the modulus of the current source density coincides with the target site. This ignores that the amplitude will be, as is almost always the case for linear methods and multiple sources, misestimated due to the unavoidable off-diagonal elements on the resolution matrix (3).
2. As the definition clearly states, the dipole localization error (DLE) is estimated from the modulus of the current source density, which means that the DLE is not a linear function of the data *d*, and thus the principle of superposition does not hold. Consequently, linear system theory, that characterizes the system by their response to (Delta like) input impulses, cannot be invocated.

Given previous theoretical flaws, it is not surprising that the DLE fails to predict the performance of an inverse solution in the presence of multiple sources. In fact it can be proved that correct localization of single sources is a trivial property of simple yet robust methods (see the work by Grave de Peralta et al. for this issue) that, we insist, are only applicable if the concentrated single source hypothesis holds.

### 5.4. Inverse Solutions and Spatial Filters

A sound approach for the inverse-problem solution in physical volumes is the estimation of spatial filters, which “filters out” the activity that arises from one special location, while trying to suppress the activity from all others. These methods that have reappeared nowadays under the name “beamformers” are very appreciated, among other things, because the solution can be computed independently for each solution point. Continuing with the original descriptions of these methods [83, 84], it was clear that minimizing crosstalk (i.e., distance to the ideal resolution matrix) between sources does not necessarily imply an optimal resolution kernel. Nevertheless, current applications suggest that the solution provided by these methods is not affected by the crosstalk. 

There are very good reasons to select a Backus-Gilbert (i.e., beamformer) method such as its adaptive properties to deal with specific noise structures [85]. However, we cannot emphasize enough that the only way to assess the estimates provided by a linear inversion procedure is to look at the resolution kernels [2, 3, 86]. The fact that we build an independent estimate for each point alone does not mean that this estimate is not contaminated by the effect of simultaneously active sources. 

In order to conclude the issue of the inverse procedure selection on a positive note, we mention that there is a sound theoretical way to select, and more importantly, to build an inverse solution. It is enough to note that infinitely-many-linear-inverse methods can be described by the equation *G* = *C* ∗ *L*′ ∗ (*L* ∗ *C* ∗ *L*′)^+^. The source *j*_est_ estimated with this method will belong to the space spanned by the columns of *C* ∗ *L*′ for both the noiseless and the noisy case. On the other hand, it is clear from (3) that the only way to change the rows of the resolution matrix (i.e., the resolution kernels) is by right transformations of the lead field. These two procedures together yield meaningful source estimates, when *C* is selected according to sound a priori information and when an appropriate right-hand transformation of the lead field is made [87].

### 5.5. Robust Methods for the Analysis of EEG/MEG Sources

The problem with the estimation of the EEG/MEG sources can be interpreted as follows. The measured data provides precise (up to the noise level) but local information. In order to know more about the whole system (i.e., the brain), we need to ascend to qualitatively higher levels corresponding to the surface maps and the 3D distribution of sources. By doing this we obtain a more complete global descriptor but probably also with a higher incertitude (if compared with the sensor data).

As it is also the case for the fMRI signal [88], in general we cannot rely on the amplitudes provided by the inverse solution to compare the neural activity at two different locations. For the same reason, ghost and lost sources appear in every reconstruction mixed with real sources. Thus, differentiating true sources from artifacts is almost impossible unless we know the real distribution. Consequently, we can say that the source distribution obtained from a single map is probably the most imprecise picture that we can have of brain activation. 

What can we do to increase the reliability of these functional images? As for a partial answer, we suggest the following points.

1. Select your inverse solution keeping in mind the previously discussed points about the spatial filters and the zero dipole localization error and consider with caution source distributions estimated from a single map (as produced, e.g., by segmentation algorithms).
2. Use source models that reduce the underdetermination of the inverse problem. Give preference to physically sound transformations that reduce the problem to the estimation of scalar fields improving the resolution kernels.
3. Compute magnitudes or figures of merit based on the temporal course of brain sources instead of the instantaneous local amplitudes and use measures that are independent of the scale factor of the intracranial signals like correlation coefficients.
4. Evaluate contrasts between experimental conditions or prestimulus versus poststimulus conditions to reduce systematic ghost and lost source effects [31, 89].
5. Compute correlations between magnitudes derived from the time course of the brain activity and behavioral measurements as reaction times [90].

## 6. Conclusion

There are many key areas that critically affect the accuracy and precision of source localization. In this paper we discussed the four key areas of EEG/MEG source imaging, namely, preprocessing, the volume conductor, and the forward and inverse problems. Notwithstanding these wide-ranging components, we emphatically direct the community to allocate attention to these key open issues. Firstly, the conductivity equally affects the forward and inverse solution thus warranting the need for actual conductivity measurements on live tissue to fill the void of these critical parameters. These future studies should accurately document their measurement setups—most especially in terms of moisture and temperature. Secondly, future modeling studies should incorporate how pathologies alter a normal, healthy head model. Lastly, it is critical to select the source model and the inverse procedure based on sound theoretical and experimental basis. 

Ultimately, we should make wise decisions to optimize elements of the model that gain the most precision and accuracy in source imaging and suppress those that contribute minimal gains in source localization. After such optimizations, how well do these future models represent their physiological counterpart, that is, the human head? As we proceed forward as a community, we should remember to highlight the shortcomings of future studies reflecting new conductivities, pathologies, source models, and so forth, to prevent any further misinterpretations of those models, while collectively building upon the contributions of past models.

## Figures and Tables

**Table 1:** A comparison of the four methods for solving Poisson's equation in a realistic head model is presented: boundary element method (BEM), finite element method (FEM), isotropic finite difference method (iFDM), and anisotropic finite difference method (aFDM).

	BEM	FEM	iFDM	aFDM
Position of computational points	Surface	Volume	Volume	Volume
Free choice of computational points	Yes	Yes	No	No
System matrix	Full	Sparse	Sparse	Sparse
Solvers	Direct/iterative	Iterative	Iterative	Iterative
Number of compartments	Small	Large	Large	Large
Requires tessellation	Yes	Yes	No	No
Handles anisotropy	No	Yes	No	Yes
